# “I think all of us should have […] much better training in ethics.” Ethical challenges in policy making during the COVID-19 pandemic: Results from an interview study with Swiss policy makers and scientists

**DOI:** 10.1186/s12910-024-01132-x

**Published:** 2024-11-15

**Authors:** Caroline Brall, Felix Gille, Caroline Schlaufer, Rouven Porz, Ralf J. Jox

**Affiliations:** 1 Ethics and Policy Lab, Multidisciplinary Center for Infectious Diseases, University of Bern, Länggassstrasse 49a, Bern, 3012 Switzerland; 2 Institute of Philosophy, University of Bern, Bern, Switzerland; 3 Digital Society Initiative, University of Zurich, Zurich, Switzerland; 4 Institute for Implementation Science in Health Care, University of Zurich, Zurich, Switzerland; 5 KPM Center for Public Management, University of Bern, Bern, Switzerland; 6 Medical Ethics, Medical Directorate, Inselspital, University Hospital of Bern, Insel Gruppe, Bern, Switzerland; 7 Institute of Humanities in Medicine, Lausanne University Hospital and University of Lausanne, Lausanne, Switzerland

**Keywords:** Ethics, Policy making, Decision-making, COVID-19 pandemic, Policy advice

## Abstract

**Background:**

The COVID-19 pandemic posed many unprecedented challenges to health care systems and public health efforts worldwide. Policy making and science were deeply intertwined, in particular with regard to the justification of health policy measures. In this context, ethical considerations were often at the core of decision-making trade-offs. However, not much is known about the actual ethical challenges encountered by policy makers and scientists involved in policy advice. With this study, we therefore aim to explore the ethical challenges during COVID-19-related political decision-making in Switzerland as perceived by policy makers and scientists involved in policy making. We also explore the role ethics advice had during the pandemic response and what can be learned for future public health crises.

**Methods:**

We conducted thirteen qualitative expert interviews with policy makers and scientists involved in decision-making on COVID-19 policy responses in Switzerland on the regional and national level. We used inductive content analysis to analyse the interviews.

**Results:**

Among the multitude of ethical challenges highlighted, interviewees perceived making trade-offs between the common good vs. the individual good and between economic welfare vs. health of the population, as well as proportionality of the policy measures, and the capacity of the public to accept uncertainty as central. Interviewees had diverging opinions on whether ethical considerations were sufficiently raised and discussed on the Swiss policy level during the COVID-19 pandemic. Among the reasons why ethics was not sufficiently discussed, they mentioned a lack of time in the fast-paced dynamic of the pandemic, ethics as a complex subject area, the interconnectedness between ethics and law, too much focus on few topics (mostly on vaccination-related ethical questions), and power relationships, such as dominance of medical professionals over ethicists. They evaluated ethics support to have been adequately present in the decision-making process, but wished for ethics training, involvement of the public in the discourse and for accompanying communication to build trust among the population for the future.

**Conclusions:**

The study provides empirical insights into the ethical considerations of COVID-19 policy making in practice in Switzerland. It can help to develop ethics assistance for future crises and inform ethical health policy and decision-making not only in Switzerland, but also in other countries.

**Supplementary Information:**

The online version contains supplementary material available at 10.1186/s12910-024-01132-x.

## Introduction

The COVID-19 pandemic posed severe ethical challenges to policy making and public health efforts worldwide, including Switzerland. In Switzerland, COVID-19 circulated early on due to its proximity to Italy where cases were first reported in Europe. Already in March 2020, Switzerland had high infection rates and the Federal Council declared an extraordinary epidemic situation in accordance with the epidemics act that lasted until 19 June 2020 [1]. Scientific input was integrated in policy making by establishing the Swiss National COVID-19 Science Task Force on April 1st 2020, which advised policy makers and informed the public about scientific findings on COVID-19 over a period of 24 months [2, 3]. Policy making and science were deeply intertwined, and ethical considerations, such as balancing public health with individual freedoms, the distribution of scarce resources, and the prioritization of vulnerable populations, were often at the core of political decision-making. Yet, not much research has been done on which ethical challenges policy makers and scientists faced and how they approached – and potentially resolved – these ethical challenges. This article addresses this gap by offering insights into the ethical challenges encountered during COVID-19-related political decision-making in Switzerland, with particular attention to the role of ethics policy advice. Ethical challenges refer to situations in which a difficult choice has to be made, or in which moral uncertainty as to the ‘right’ way forward exists [4]. With ‘ethical considerations’ we refer to the broader reasoning to address ethical challenges. With ethical issue we refer to a specific situation or topic that inherently involves questions of right and wrong and the normative question of what ought to be done. By drawing from expert interviews conducted with policy makers and scientists, we aim to explore the ethical issues and challenges in the decision-making process and shed light on how these challenges were met.

Public policy decision-making is an inherently political process, characterized by the struggle for influence of different actors [5, 6]. The systematic weighting of fundamental ethical values does not necessarily determine the outcomes of political decision-making, as decisions are also influenced by interests, power, and ideologies. Nonetheless, ethics is relevant at all stages of the policy making process [7]. While policy analysis is concerned with what governments do, why they do it, and what difference it makes [8], ethics examines what ought to be done. This includes the examination of what governments ought to do and what ethical principles guide policy making [9].

We understand ethical reasoning in public policy as a process by which policy makers and policy advisors identify, analyse, and weigh the ethical issues of proposed policies. This process involves considering fundamental ethical values of the matter, such as health maximisation, autonomy, beneficence, non-maleficence, justice and proportionality when tackling a pandemic, as well as other societal values such as accountability, transparency, effectiveness and sustainability [10]. Ideally, potential impacts of policies on diverse stakeholders are assessed, aiming to choose a course of action in public health policy that upholds ethical standards, promotes fairness, and contributes to the overall well-being of individuals and communities within the context of a given societal framework.

Given the distinct ethical challenges of pandemics, lessons from the COVID-19 pandemic can be learned for future pandemic outbreaks, especially with regard to what ethical challenges policy makers faced, how the ethical challenges were dealt with during COVID-19-related policy making and what kind of ethics assistance would be useful for future public health crises. Our study seeks to contribute practice-based insights that can inform future decision-making during public health crises and guide the development of health policy advice involving ethics.

## Methods

We selected a qualitative, explorative approach for our study and conducted expert interviews with policy makers and scientists involved in decision-making on COVID-19 policy responses in Switzerland.

### Data collection

The main author (CB) conducted expert interviews with Swiss policy makers and scientists involved in COVID-19 policy making from February until May 2022. Both scientists and policy makers were interviewed, as they were the main actors in COVID-19 related policy making. While scientists had an advisory role through, e.g., the Swiss National COVID-19 Science Task Force, policy makers were directly involved in decision-making and its implementation, either as politicians or in public administration. All participants were professionals over 18 years of age, and of both genders, who meet the following inclusion criteria: being a policy maker or scientist involved in policy advice on COVID-19 matters. The interviewees were selected according to purposive sampling of maximum variation based on the interviewee’s function (policy maker or scientist), level (national, cantonal), and region (East-Switzerland, Central-Switzerland, West-Switzerland) (see results). Scientists were selected who were members of the Swiss National COVID-19 Task Force, and policy makers who held a key role in public administration and politics, e.g. as heads of divisions of the Federal Office of Public Health, cantonal chief medical officers, or members of the National Council. Interviewees furthermore referred to colleagues, so that we also employed a snowball sampling approach. Overall, we contacted eighteen potential interviewees by email including a cover letter and sent a reminder letter two weeks later. Five potential participants declined the invitation due to lack of time or did not respond. At thirteen interviews, thematic saturation was reached.

Based on our research questions and an initial literature review, CB developed a semi-structured interview guide to guide the explorative data collection. By means of a semi-structured interview ‘subjective theories’ about a set of themes can be iteratively reconstructed, which we aimed to achieve with this study [11]. The interview guide was developed for this study and involved questions on which role ethical considerations played in the interviewees’ decision-making and which form of ethical policy advice was and would be useful for them in the future (see supplementary material). The second part of the interview guide addressed the topic of technology and health data use, but was not considered for this article and will be addressed in a separate publication. The interview guide deliberately entailed few main and sub-questions, so that the interviewer and interviewees were given space and flexibility to follow topics as the conversation unfolded. CB conducted the interviews via video-supported telephone interviews (using the software Zoom). After participants gave verbal consent, the interviews were recorded, transcribed verbatim and de-identified.

### Data analysis

We analysed the data by applying content analysis in line with Mayring [12]. We categorised and summarised the data according to emerging categories and two levels of sub-categories. In a first step, all interview transcripts were read in full to gain an overall impression of the emerging concepts. In a second step, three authors (CB, RP, RJ) independently coded categories and sub-categories of one interview and compared their outcomes. Here, the overall categories were reached deductively, since they followed the themes touched in the semi-structured expert interviews. The sub-categories, however, were derived inductively and new concepts could be identified. Two external researchers validated the identified categories and sub-categories. Afterwards, the CB coded categories and sub-categories in each interview. After a thorough re-analysis, the results were discussed and validated by the authors.

## Results

We conducted thirteen semi-structured explorative interviews with policy makers and scientists involved in COVID-19 policy making between February and May 2022. In qualitative interview studies thematic saturation usually emerges between six and twelve interviews in homogeneous groups [13]. Even though policy makers and scientists are heterogeneous groups, their evaluation of COVID-19 policy making did not diverge extensively. The analysis of interviews showed that thematic saturation was reached at thirteen interviews. The interview length varied between 22 min and 52 min with a mean of 38 min. We involved interviewees from all parts of Switzerland, on cantonal and national level and who held different roles and functions in policy making and scientific advice. Table 1 shows an overview of the interviewees, including their role, gender (identified by the authors without letting interviewees self-identify, using a binary understanding of gender), level of action and geographical region (as defined by Canton). The interviewed scientists were from the fields of communication science, data science, economy, epidemiology, ethics; and among the interviewed policy makers were cantonal chief medical officers, division heads of the Federal Office of Public Health, members of the National Council, members of cantonal crisis units and staff members of the Federal Department of Home Affairs.

**Table 1:** Interviewee characteristics

Characteristics	% (*n*)
**Role**	
Scientist	46.2 (6)
Policy maker	53.8 (7)
**Gender**	
Male	76.9 (10)
Female	23.1 (3)
**Level**	
National level	84.6 (11)
Cantonal level	15.4 (2)
**Canton**	
Berne	46.2 (6)
Geneva	7.7 (1)
Grisons	7.7 (1)
Ticino	15.4 (2)
Valais	15.4 (2)
Zurich	7.7 (1)

Five key-themes emerged from the interview data: (1) perceived ethical issues during the COVID-19 pandemic, (2) the role of ethical considerations in policy making, (3) perceived actors of ethical considerations and critical evaluation, (4) evaluation of the sufficiency of ethical considerations during the COVID-19 pandemic, and (5) desired ethical support. These five key themes can be grouped in two broad categories: COVID-19 policy making and the self-reflection of interviewees. Table 2 provides an overview of the key themes.

**Table 2:** Overview of key themes

COVID-19 policy making			Self-reflection - Interviewees’ evaluation	
**Perceived ethical issues during COVID-19 pandemic (key themes)**	**Role of ethical considerations in policy making process**	**Perceived actors**	**Evaluation: sufficiency of ethical considerations**	**Desired ethical support**
● Making trade-offs between the common good vs. the individual good ● Acting in line with the principle of proportionality ● Making the trade-off between economic welfare vs. health of the population ● Capacity of the public to accept uncertainty	● Diverging perceptions about the role of ethical considerations in policy making: central role vs. subordinate ● Ethical considerations were made implicitly when addressing values which guided decision-making	● Key actors: ethics sub-group of the Science Task Force; individual ethicists working in academia ● Secondary actor: National Advisory Commission on Biomedical Ethics	● Diverging opinions whether ethical considerations were sufficiently addressed on policy level: sufficient vs. not sufficient Hindering factors: ● Lack of time ● Ethics as a complex subject area ● Interconnectedness between ethics and law ● Narrow focus on few topics (e.g. vaccines) ● Dominance of medical professionals over ethicists	For future public health crises: ● Ethics trainings for those involved in policy making ● Need for ethical support to help with dilemmas and excessive demands ● Involvement of the public in discourse ● Need for accompanying communication to build trust among the population

## Ethical issues in COVID-19 policy making as perceived by policy makers and scientists

All interviewees perceived that there was a multitude of ethical challenges arising from the COVID-19 pandemic, describing it as a “huge problem” (P08) for policy makers, scientists and society in general. It was stressed that pandemics expose conflicts of values more frequently and that solving conflicts of values is not part of the usual public discussions:

*“[…] a pandemic is such a holistic systemic crisis that really does a stress test of the entire society. It means that it confronts us to conflicts of values everywhere. […] A lot of the controversies*,* a lot of the public discussions were actually there due to the fact that it is infrequent that we are placed so repeatedly in front of conflicts of values altogether on a daily basis almost.” (P03)*

Several ethical issues during the COVID-19 pandemic were mentioned on two levels: (i) ethical issues arising in health care facilities such as hospitals and long-term care facilities, and (ii) on the public health and societal level. Regarding the former, the need to fairly allocate resources in hospitals, such as ventilators and hospital beds, led to the development of triage criteria early on during the pandemic [14]. Other issues involved postponing elective interventions in hospitals, the increased relevance of advance care planning in individual care settings, reduced visiting rights in long term care facilities and restrictions for residents to leave such institutions. Most interviewees referred to issues on the public health and societal level. One interviewee contextualised decisions made during a pandemic as a contract between the collective society and individuals.

*“The contract is as follows: the authorities ask all of us to make choices*,* to make different choices than we would have otherwise*,* to make sacrifices*,* to take burdens*,* sometimes to expose ourselves to risks. […] Think about nurses and nursing home staff. Sometimes we are asked to expose ourselves to risk for the common good. In return*,* the authorities promise to do all they can to protect three things: to protect our lives and physical integrity*,* our health—that’s the most visible part but it’s not the only part. They also promise implicitly or explicitly to do all they can to protect our rights and to protect our means of subsistence. […] The interdependence of everyone becomes extremely visible in the face of a contagious disease.” (P03)*

We furthermore asked interviewees what they perceived as the biggest ethical challenge of policy making during the COVID-19 pandemic. Most interviewees indicated that making trade-offs between safeguarding the common good vs. safeguarding the individual good and acting in line with the principle of proportionality was the biggest ethical challenge in their work as policy makers or scientists involved in policy advice. Determining what measures could be implemented and anticipating whether they will be accepted and supported by the public was mentioned as particularly challenging. Other interviewees highlighted the trade-off policy makers had to make between protecting the economic welfare vs. protecting the health of the population:

*“You want to both save the economy and you want to save lives*,* but sometimes you can’t do that too at the same time. To save more lives you have to put a break on economic activity. Ethically*,* this is a hugely difficult choice.” (P01)*

Health equity was perceived as a key underlying concept within the ethical considerations made:

*“There were a lot of ethical concerns about policy measures reaching or not reaching vulnerable people.” (P06)*

In view of these trade-offs that needed to be decided upon during the COVID-19 pandemic, the capacity of the public to accept uncertainty was mentioned as a key challenge for successful pandemic management. Interviewees stressed the need for public trust in the government to effectively address the pandemic, make ethical decisions, and acknowledge any mistakes made by policymakers in the process.

*“A concept that was very*,* very important to me from the ethical perspective*,* and this is not connected with a decision*,* but with the management of the crisis*,* is the ambivalence capacity of the population. That is the question*,* how can the population also deal with mistakes that we make in the management of the crisis […].” (P10)*

## The role of ethical considerations in the policy making process

The interviewees perceived the role ethical considerations played in policy making differently: While some expressed that ethical considerations played a central role in policy making taking place when discussing trade-offs, others deemed ethical considerations as subordinate in policy making.

“*Even though many people would not call them ethical issues*,* but would call them trade-offs*,* they were very visible. […] These trade-offs were very much part of decision-making […].” (P03)*

*“The government*,* the task force or experts have addressed ethical issues sporadically. However*,* it did not seem to me that there was a particularly strong ethical debate.” (P01)*

Those that saw ethical considerations as subordinate framed them as solely focussing on individual responsibility to avoid infections.

*“[Ethics was] rather subordinate. The discourse was rather technocratic and*,* not least*,* pushed ethical considerations into the area of individual responsibility.” (P13)*

While some interviewees specified that decisions were not explicitly assessed from an ethical perspective during policy making, others saw the ethical issues addressed, but evaluated them as sometimes unfruitful and misguided and hindered the practical realization of policy measures:

*“I think there was always a discussion about them [ethical considerations]. In some cases*,* they really hindered us from doing what was needed because I think that the discussion was misguided*,* misplaced or misunderstood.” (P06)*

One interviewee working in the government, emphasized that ethical considerations were not taking place explicitly in decision-making processes, but were rather considered implicitly when addressing values which guided decision-making:

*“That we would have followed an ethical checklist for every measure… I have to say quite clearly*,* no*,* but it was part of the work we did*,* yes. And I think ethical standards always have to do with a value system. I think that comes automatically*,* simply because we all have values and represent values. Of course*,* that also comes into play somewhere.” (P09)*

An explanation for the insufficiency of ethical considerations in policy making mentioned by some interviewees was that sufficient time for extensive discussions was lacking during times of crisis, where decisions must be taken rapidly:

“*But ethics also involves weighing up the arguments until I reach a decision. I can’t do that in a crisis. In a crisis*,* I have to make tremendous decisions in an incredibly short time. And then it’s not ethical questions*,* it’s straightforward. It’s sort of A or B. I have to make [a decision].” (P10)*

## Perceived actors of ethical considerations

Interviewees were asked who they perceived as the key actors of making ethical considerations during COVID-19 policy making in Switzerland and critically evaluated them. The interviewees named the ethical sup-group of the Science Task Force and individual ethicists working in academia as the key actors. The National Advisory Commission on Biomedical Ethics was mentioned as a secondary actor. In general, the interviewees evaluated the work of the ethical sup-group of the Science Task Force as helpful.

*“It was useful. Now I can speak from the Task Force. There were people with an ethics background*,* there was a whole subgroup of the Task Force with an ethical and legal background. They were tremendously important and influential in a lot of the discussions. I don’t think generally ethics was insufficiently present.” (P01)*

Some, however, wished for a stronger positioning of the Science Task Force with regard to ethical issues. It was mentioned that this might have not taken place due to the member setup and selection of the Science Task Force.

*“Everyone has tried it a little bit. Of course*,* the ethics side was represented in the Science Task Force. Sometimes we would have liked to see a stronger position with regard to ethical questions that arose*,* but somehow there was a feeling that a certain selection was taking place in the participation in this Science Task Force*,* and that a truly comprehensive ethical evaluation was not taking place.” (P07)*

The National Advisory Commission on Biomedical Ethics supported the ethical analysis, but was not perceived as an actor to actively make decisions on ethical questions:

*“But there were always people in the whole phase who called and said: “But now ask the ethics committee. In the sense of a court*,* they know what is ethical*,* don’t they?” And those were also exciting conversations with [member of the National Advisory Commission on Biomedical Ethics]*,* who said: “We don’t judge for you*,* we can only give you the basics. And we can try to judge whether the decision-making processes and the principles for the decisions are correct or not.” (P10)*

Authorities and politicians were perceived critically for having not detailed the ethical aspects of certain public health measures to condemn the COVID-19 pandemic nor pointed out an ethical strategy. One interviewee depicted that delineating the ethical aspects would support the social acceptance of measures.

*“Yes*,* we want to have socially acceptable measures*,* but the ethical aspects have not been described in such detail by the authorities and politicians*,* and that surprised me. […] The public health response or pandemic management has always been very much of the moment and not based on ethical principles or a strategy.” (P04)*

A reason why this was perceived critically was that definitions of ethical reasoning and decision-making diverged and that it was challenging for different actors working in the field of ethics to find out where, when and in what way ethical deliberation should happen.

*“I think everyone has tried to make an ethical assessment based on what they understand by ethical decision-making*,* and that is certainly something different from what I understand by it than what you understand by it necessarily. And so it has gone from politics to administration*,* to science*,* and everyone has tried to classify it somehow for themselves.” (P07)*

Interviewees furthermore raise the argument that ethical discussions often tend to revolve around trade-offs, which are seen to be core to the political process. It was pointed out that policy makers should receive ethical guidelines, but ultimately have to decide themselves and take responsibility for it, which *“cannot be taken away”* (P10).

*„The difficulty*,* which is certainly there*,* is that when it comes to ethics*,* one very quickly finds oneself in these discussions about weighing things up*,* which politics not unjustly claims for itself.“ (P12)*

In general, interviewees perceived the difficulty that actors undertaking ethical deliberation were not sure where to position themselves, as one interviewee put it:

*“[…] perhaps it was difficult to find out where ethics should position itself.” (P04)*

## Evaluation: sufficiency of ethical considerations during the COVID-19 pandemic

The interviewees had diverging opinions whether ethical considerations were sufficiently raised and discussed in Swiss policy making during the COVID-19 pandemic. While some thought that ethical considerations were sufficiently discussed, others perceived them as not being sufficiently discussed in policy making. Among the reasons why ethics was not sufficiently discussed, they mentioned a lack of time in the fast-paced dynamic of the pandemic, ethics as a complex subject area, the interconnectedness between ethics and law, too much focus on few topics (mostly on vaccination-related ethical questions), and power relationships, such as dominance of medical professionals over ethicists.

*“It needs a kind of instant ethics*,* and ethicists are sometimes very careful to analyse and research everything in detail*,* and in a profound way*,* including the pros and cons*,* and sometimes it remains very abstract. And in such a situation*,* however*,* you may need an assessment very quickly*,* based on very little knowledge*,* and something very pragmatic*,* that is certainly the challenge.” (P12)*

One topic which should have been addressed more in depth during the decision-making process was the distinction and relationship between individual freedoms and individual duties. One interviewee expressed that such discussion could have provided a better basis for solidarity within the population.

*“I completely missed a discussion of what individual rights are based on. The focus was largely on individual freedoms. However*,* the fact that these are only possible if duties are also fulfilled was largely left out. An honest discussion of this could perhaps have contributed to more solidarity and community spirit.” (P13)*

## Desired ethical support

Interviewees expressed their desired ethical support both retrospectively during the COVID-19 pandemic, as well as prospectively regarding future pandemics. According to the interviewees, it would have been useful if a specific body, such as the Federal Commission for Pandemic Preparedness and Response, had developed a comprehensive list of potential ethical issues related to a pandemic prior to an outbreak. This could have included lists of potential dilemmas, suggestions on how to address them, and a set of guidelines to facilitate decision-making. In addition, interviewees proposed a crisis committee that comes in in the event of an emergency to examine the aspects of pandemic preparedness, similar to the established Science Task Force. They raised that the ways of functioning between science and politics only evolved with time during the pandemic and need better explication in future crises. Another resource policy makers regarded as helpful to tackle the ethical challenges of the COVID-19 pandemic was the National Advisory Commission on Biomedical Ethics and individual ethicists, who were approached for exchange on certain topics. All actors can function as resources during future public health crises.

Relating to an ethical strategy, some interviewees mentioned that it would have been helpful to explicate the values according to which public health measures should have been implemented:

*“So we actually missed the opportunity to think about what the values are that need to be protected and how to weigh up allowing the health system to be overburdened: can we allow such record high excess mortality or are we socially responsible to prevent it*,* and how do we want to do that?” (P04)*

*“How can we prepare in such a way that we put certain values down on paper in case of a crisis? And how can we implement these guidelines as quickly as possible in the event of a new crisis?” (P07)*

Some interviewees believed that the discipline of ethics could have provided a more pragmatic decision-making framework and concrete suggestions, rather than merely engaging in an ongoing discourse. In addition, an argument-based exchange was wished for, rather than clashing opinions of “*I am right and you are not right*” (P02). Direct, personal exchange with ethicists was valued by policy makers as highly beneficial to internally discuss ethical concerns in a timely manner before making the discussions public. Yet, it was mentioned that such direct, personal exchanges might be difficult to scale. Having an informal network comprising ethicists was expressed as one way. A tool that could facilitate ethical discussions is an online chat (e.g. using the instant messaging software Slack), which would allow for timely exchange for everyone involved and being able to stay up to date.

*“If the whole thing were to happen on a Slack channel*,* for example*,* maybe. Just as an example. Then I could follow this discussion*,* everyone could follow this discussion and if necessary also participate and then take their part. But one could have discussed relatively quickly. And for me*,* that’s a bit of a wish*,* that we have these digital communities in which we can discuss together very quickly. That it is visible for everyone in this community*,* but not necessarily public. […] And then you could immediately have the conversation and somehow agree on what is good and what is bad. And the others*,* they come maybe two hours later and they see this discussion. But everyone is in the loop. And you don’t have to somehow say*,* ‘We’re organising a meeting. […]’ Because that’s not the dynamic either*,* it’s much too slow.” (P02)*

### Ethics support in future public health crises

With regard to future pandemics and other public health crises, the interviewees wished that all those involved in policy making – including scientists – should receive training in ethics, for instance offered through universities or universities of applied science, to build better knowledge in ethics in the long term:

*“I think all of us should also have a training in universities and universities of applied science; a much better training in ethics. […] I think that that could go a long way also as a long-term solution.” (P06)*

They evaluated ethical assistance as urgently needed in times of crisis in order to help policy makers overcome dilemmas and overwhelming situations in decision-making. Furthermore, the interviewees expressed that the public should be better involved in the ethical discourse to include the societal view on such topics in a representative way:

*“[…] the ethical discourse*,* the social discourse*,* that was not managed to be captured in a representative way*,* which is probably also difficult or maybe even unsolvable*,* but nevertheless it must happen on a larger scale next time.” (P07)*

Lastly, accompanying communication about who the ethics experts are was regarded as necessary to build trust among the population, since many experts were suddenly in the public focus, but the population was not informed about their influence and position.

*“[…] suddenly a lot of people came like a phoenix from the ashes and positioned themselves*,* nobody knew them*,* we didn’t know whether we could trust them or not. The population somehow didn’t know what to do with them. And I really believe that trust has to be built up continuously over the long term*,* so that the population knows that if something happens*,* I will see the person again and the trust is there that I will follow what the person says.” (P07)*

## Discussion

The purpose of this research was to explore the ethical considerations during COVID-19 policy making that took place in practice in Switzerland through explorative interviews with policy makers and scientists. An added value of our research is that it empirically assesses the ethical challenges that appeared in COVID-19 policy making, focusing not only on the topics of ethical issues – the content of the ethical debate –, but also on the challenges of integrating ethical reasoning in the policy process. While there is academic literature about the normative role of ethics in public policy making [15], the practical role ethics may play in the public policy making process has been elucidated insufficiently so far. With regard to ethics in the public policy making process in relation to the COVID-19 pandemic, we only found few relevant articles [16–19]. Pykett et al. observed that ethical considerations were frequently discussed in the public debate during COVID-19, yet have not received much attention in policy making and that understanding of ethics advice processes in different countries is lacking [17]. Our article thus aimed to elucidate these questions for Switzerland. We will discuss the results of our research by addressing the ethical issues that emerged in COVID-19 policy making, the role of ethics in the policy decision-making process, and what can be learned for future crises with regard to ethics support.

The key ethical challenges as perceived by the interviewees included making trade-offs between common vs. individual good and economic welfare vs. health of the population, proportionality of policy measures, and public capacity to handle uncertainty. These themes emphasize the depth and breadth of ethical dilemmas encountered in policy making during the pandemic and are in line with the themes found in the recent academic literature listing the ethical issues appearing during the pandemic [20, 21]. The challenge of having to make trade-offs between the competing values of common good vs. individual freedoms is not new and known as a dilemma at the heart of pandemic policy making and public health in general. Our findings emphasize that these trade-offs were also perceived in the real-world setting with increased tension during the COVID-19 pandemic. A global interview study with 39 bioethics experts found that the experts mentioned ethical issues that arise on a health care facility level rather than a public health and societal level [21]. This could be explained by the fact that many of the interviewees were clinicians or working in the clinical field and thus focused on their practical experiences. However, this focus on ethical issues on a health care facility level is also reflected in other publications published since COVID-19 [22–25]. In this research, we explicitly asked interviewees about public health issues. Yet, solving issues on the public health level is often highly complex – also described as a ‘wicked problem’ [26] – and thus most interviewees might mention issues arising at the micro level in health care facilities when not asked directly about the public health level.

The analysis revealed that interviewees had diverging opinions as to whether ethical considerations were central or subordinate during decision-making on COVID-19 policies in Switzerland. This discrepancy between the existence of ethical challenges and that some interviewees deemed the ethical considerations as sub-ordinate during decision-making on COVID-19 policies suggests that it was neither properly defined what was understood as being an ethical issue, nor how to address these ethical issues in the policy making process. This polarized view furthermore underscores the subjective nature of ethical evaluation and the challenges in assessing the adequacy of ethical deliberation. We suggest that future crisis management can prepare for this by clearly defining what ethical issues are at stake and to integrate a process for conducting ethical considerations in the policy making process. In line with this, the interviewees flagged the need of a clearly defined national ‘ethical strategy’ to address the arising ethical issues. Such ‘ethical strategy’ could lay out the values that guide the decision-making (for instance maximizing health of the population as a primary value), the role of the ethics advisor and the process how ethical considerations will be assessed. Instead of a one-size-fits-all-approach ethics support needs to be context-sensitive, providing a more explicit compass that can be applied in urgent, high-stakes contexts [26]. Our study moreover highlights whom interviewees perceived as the main actors in conducting ethical considerations, identifying both institutional and individual contributors to the ethical discourse. The pivotal role of ethics advisory groups, such as the ethics subgroup of the Swiss National COVID-19 Science Task Force, aligns with recommendations to integrating ethical expertise in pandemic preparedness and response efforts in an institutionalized way [18, 19, 27]. Yet, ethics expertise was also oftentimes sought in an ad hoc way, where individuals of ethics advisory groups were contacted for ethics advice. Given the fast-paced environment during the pandemic this seemed as a pragmatic way to attain support in ethical questions, provided that the individuals also had an institutionalized role in an advisory group in order to not erode public trust [19]. However, while this ad hoc approach was pragmatic, it raises questions about the transparency of such ‘internal discussions’. Interviewees’ opinions were contradictory with regard to having ‘internal discussions’ via digital software tools that are not visible to the public and the request to involve the public more in the ethical deliberation on appropriate public health measures. While it might be understandable that discussions need to take place among experts in a closed setting first, transparency about the points discussed and the outcomes is essential for both public legitimacy and the ethical robustness of decisions made under crisis conditions. Ensuring transparent communication about such engagements and the outcomes could enhance the public’s confidence in the decisions made. An institutional role could furthermore serve as a guarantee of the ethical advisor’s credibility and accountability.

In contrast to the polarized view on whether ethical considerations were central or subordinate during decision-making on COVID-19 policies in Switzerland, most interviewees estimated that ethics support was adequately present in the COVID-19 decision-making process. They however proposed further methods to foster such ethics support in the future, such as trainings, ongoing communication channels via the software ‘Slack’ or applying checklists and flow charts to answer ethical questions. Our research thus underscores the perceived need for enhanced ethical support and training among policy makers and scientists, suggesting a gap between the theoretical ideals of ethical reasoning and their practical implementation. This gap points to potential areas for improvement, such as the development of more structured ethical frameworks and guidelines that can be readily applied in the fast-paced environment of public health crises [28]. Furthermore, the varied perceptions of the sufficiency of ethical considerations in policy decisions highlight the subjective nature of ethical evaluation and thus may hint towards the importance of establishing clear, transparent criteria for ethical deliberation. The terms ‘ethics’, ‘ethical decision-making’ and ‘ethical reasoning’ were deliberately left undefined prior to the interviews, allowing interviewees the freedom to interpret and articulate their own understanding of these concepts. However, this may also explain the variation in perceptions among the interviewees. While much research has been done since the COVID-19 pandemic about the role of science in policy making [2, 3], it is noteworthy that only few papers address how ethics support and advice – which was key during the pandemic given the multitude of ethical challenges – was integrated in policy making [16, 17, 27]. Wilson et al. suggest four ideal types of ethics advice for public policy which are based on whether an individual or public interest perspective is taken and whether recommendations are made or not: The ‘pure ethicist’ takes an individual perspective and refrains from making specific recommendations; the ‘advocate’ also takes an individual perspective but makes recommendations and engages in decision-making processes; the ‘ethics arbiter’ takes a public interest perspective and does not make recommendations but aims to support policy makers to understand relevant ethical values and reasons and thus to enable them to reach their own decision; and the ‘critical friend’ also takes a public interest perspective but makes recommendations for certain policies [27]. During the COVID-19 pandemic in Switzerland – as for scientific support in general [2] – it was not clearly established from the beginning which role ethicists took on or were officially asked to take on. In the course of the pandemic the role and tasks of the Swiss National COVID-19 Science Task Force in scientific policy advice became clearer and made a “clear distinction between the political decision-making process on the one hand and the presentation of scientific findings during the advisory process and for the attention of the public on the other: scientists provide advice and policymakers make decisions” as stated in the Final Report of the Swiss National COVID-19 Science Task Force [29]. Scientific advice thus was made according to the type of ‘ethics arbiter’ (or rather ‘science arbiter’ in this case). The interviewees however saw the role of ethics advice either to take the ‘ethics arbiter’ or the ‘critical friend’ role: Most interviewees expressed that ethics support should take a public interest perspective and should identify underlying values and reasons (‘ethics arbiter’). Policy makers should thus be enabled to make their own decisions. Other interviewees would have liked to receive more specific recommendations (by means of ‘critical friend’ support).

The interviewees furthermore frequently mentioned that it would have been relevant to involve the public more in the ethical deliberation on COVID-19 measures and related decision-making. For instance, mini-publics or citizen juries could be employed as effective means to engage a broader spectrum of society in these critical discussions. Such participatory approaches would not only democratize the decision-making process but also ensure that diverse perspectives and values are considered [30, 31]. By making values and ethical considerations more explicit and fostering public deliberation on these topics, societal acceptance of the measures could be enhanced [32]. Increased transparency to contribute to public deliberation and inclusivity in the ethical deliberation process would likely lead to a higher degree of public trust and cooperation [32, 33]. Consequently, involving the public in a structured and meaningful way could bridge the gap between policy makers and citizens, ensuring that public health measures reflect a collective ethical standpoint and are more widely supported by the public. In addition, policy makers and scientists could foster trust through openly communicating uncertainty and the possibility of making mistakes [34].

### Limitations

A limitation of this study is that findings are not directly generalizable to other contexts as the study focus is Switzerland. It also does not allow for generalizable statements for all policy makers and scientists involved in Swiss COVID-19 policy making. Yet, the qualitative nature of the research provides in-depth insights into the challenges of ethical reasoning during COVID-19 that are otherwise difficult to obtain. Future research could address this gap by exploring ethical challenges in diverse cultural and political contexts. The effectiveness of different forms of ethical support could also be investigated, such as training programs, different forms of ethics advisory, or methods for enhancing policy makers’ knowledge and skills in ethical analysis and balancing.

## Conclusion

This study enriches the existing literature on public health ethics by providing empirical insights into the ethical considerations of COVID-19 policy making in Switzerland. Based on the findings we suggest that future crisis management should entail an ‘ethical strategy’ that defines (a) the underlying values guiding and shaping decision-making on trade-offs, (b) the role or self-understanding of the ethics advisors, (c) the process how ethical reasoning should be conducted and (d) involves the public in ethical deliberation.

Justifying health policies based on ethical values provides numerous benefits: it strengthens the moral rationale for governmental actions, enhances public support, and ensures that policies are made with consideration for a broad spectrum of ethical values. Results of this interview study can help to develop ethics assistance for future crises. Potentially, it can inform health policy making not only in Switzerland.

## Electronic supplementary material

Below is the link to the electronic supplementary material.

Supplementary Material 1

## Data Availability

The participants of this study did not give written consent for their data to be shared publicly, so due to the sensitive nature of the research interview transcripts are not available.
