# Supplementary material for: “I think all of us should have […] much better training in ethics.” Ethical challenges in policy making during the COVID-19 pandemic: Results from an interview study with Swiss policy makers and scientists
Source: BMC Med Ethics. 2024 Nov 15;25:129. doi: 10.1186/s12910-024-01132-x (PMC11566081; doi:10.1186/s12910-024-01132-x)
Supplement: Supplementary file 1 — Supplementary Material 1 [file 12910_2024_1132_MOESM1_ESM.docx]

**Interview guide**

Ethical challenges in policy making during the COVID-19 pandemic and the use of technology

Expert interviews with policy makers and scientists involved in COVID-19 policy making

| Topic | Main questions | Sub questions |
| --- | --- | --- |
| Ethics and policy making | When you think about all the ethical problems during the Covid-19 pandemic, what comes to your mind?  *Ethics assistance:*  Based on what you experienced regarding decision-making during the pandemic, was or would have ethics assistance been useful and in which form? | What were the key ethical questions or issues during the pandemic in your view?  How did you perceive the role ethical considerations play or played in decision-making activities concerning Covid-19 policy responses?  By whom and how were these ethical considerations made?  Were these ethical considerations sufficient in your view?  Do you think ethics assistance would be useful for tackling future pandemics? If yes, in which form? |
| Technology and health data use | *During this pandemic, digital health apps were used in this form for the first time in Switzerland: The SwissCovid app for contact tracing and the Covid Cert app for showing Covid certificates.*  Was the use of such digital health apps (which could be used by the public) important as part of the pandemic response in your view?  Do you think the use and application of such digital information technologies could have been extended? For example, to collect anonymous health data for research purposes? | How do you evaluate the use of such apps in the context of pandemic response?  How do you expect the future application and usage of such technologies in the field of health? |
| Conclusion | Do you have any other comments or suggestions for policymaking in times of COVID-19 or future pandemics? |  |
